# Low-Temperature Methane Catalytic Combustion on a New Pt-Based Catalyst Confined in a MnO_2_/Al_2_O_3_ Carrier

**DOI:** 10.3390/molecules31111942

**Published:** 2026-06-03

**Authors:** Xiaoyi Zeng, Ruikun Zhang, Huabing Wu, Xianbing Xiang

**Affiliations:** 1 Intelligent Engineering College, Chongqing Electric Power College, Chongqing 400030, China; zengxiaoyi@cqepc.edu.cn (X.Z.);; 2 Sichuan Provincial for Rare Earth & Vanadium-Titanium Based Functional Materials, Sichuan University, No. 24 South Section 1, Yihuan Road, Chengdu 610065, China; 3 Key Laboratory of Advanced Special Materials & Technology, Ministry of Education, Chengdu 610065, China; 4 School of Materials Science & Engineering, Sichuan University, Chengdu 610065, China

**Keywords:** hierarchical confinement, Pt-MnO_2_ interface, oxygen vacancy, methane catalytic combustion, stability

## Abstract

In this study, a hierarchically confined Pt/MnO_2_–meso-Al_2_O_3_ catalyst with 0.5 wt% Pt loading was synthesized via a precipitation method using MnO_2_ as a promoter and mesoporous Al_2_O_3_ (m-Al_2_O_3_) as a support, and its methane catalytic combustion performance and structure–activity relationship were systematically investigated. The results demonstrate that the 0.5 wt% Pt-loaded Pt-MnO_2_/m-Al_2_O_3_ catalyst achieved 90% methane conversion at 236 °C. The enhanced performance is attributed to three synergistic mechanisms: (1) Pt doping induced lattice contraction in MnO_2_ (XRD revealed a 0.03 Å reduction in the (001) interplanar spacing), which facilitated the formation of Mn^3+^–oxygen vacancy pairs (XPS indicated a Mn^3+^- content of 79.87%); (2) the MnPt_3_O_6_ interfacial structure (HAADF-STEM confirmed lattice spacings of 0.21 nm) accelerated oxygen species cycling, with the 0.5 wt% Pt-loaded catalyst for lattice oxygen desorption capacity (O_2_-TPD) increasing by 54% compared to undoped samples; (3) the mesoporous m-Al_2_O_3_ carrier provided effective confinement, achieving a high specific surface area (27.6 m^2^/g) and sub-nanometer Pt dispersion (particle size < 2 nm). Under conditions of 1000 ppm CH_4_ and a space velocity of 30,000 h^−1^, the catalyst maintained a methane conversion rate of 98.2 ± 0.5% during continuous operation for 300 h. Post-cycling characterization revealed a stable crystalline structure (XRD full width at half maximum of 0.35° ± 0.02°) and grain size (15.5 ± 0.5 nm), confirming its robustness for industrial applications. This study provides theoretical and experimental foundations for the rational design of highly efficient catalysts for low-concentration methane elimination. For comparison, a Co-doped catalyst (1.0 wt% Co–MnO_2_/Al_2_O_3_) was also prepared, which exhibited significantly lower activity (T_90_ = 251 °C), underscoring the unique role of Pt in the confined architecture. This study provides theoretical and experimental foundations for the rational design of highly efficient catalysts for low-concentration methane elimination.

## 1. Introduction

Methane catalytic combustion has emerged as a pivotal technology for mitigating anthropogenic emissions of methane—a potent greenhouse gas with 84-fold higher global warming potential than CO_2_ over 20 years [1]. This process enables efficient elimination of low-concentration methane (<10%) from natural gas systems, coal mines, and landfills, simultaneously recovering energy [2]. Despite its promise, industrial implementation faces fundamental challenges rooted in methane’s high C–H bond energy (434 kJ/mol) and sluggish kinetics under mild conditions [3].

Conventional Pt/Al_2_O_3_ catalysts exhibit superior activity but suffer from irreversible deactivation via sintering (>500 °C) and hydrothermal poisoning [4]. Recent studies reveal that Pt nanoparticle coalescence accelerates under cyclic redox conditions, with >50% activity loss within 100 h [5]. While core-shell architectures (e.g., Pt@SiO_2_) enhance thermal stability, they inevitably sacrifice active site accessibility. For instance, Lei et al. [6] demonstrated that 2D-confined Pt catalysts maintained 90% dispersion after aging at 800 °C but showed 40% lower TOF than unconfined counterparts due to diffusion limitations. Similarly, Xu’s Pt@MnO_2_ core-shell design [7] achieved 300 h stability at 500 °C, but required a temperature higher than 300 °C to reach 90% methane conversion (T_90_ > 300 °C), constrained by thick MnO_2_ shells (>5 nm) that impede oxygen mobility. (Note: T_90_ is commonly used as a benchmark in catalytic combustion to avoid mass-transfer limitations near equilibrium; it reliably reflects intrinsic activity.)

To reconcile activity–stability trade-offs, multifunctional interfaces have gained traction. Zhang et al. [8] pioneered MnO_2_-coated Pt catalysts where oxygen vacancy (O_v_)-rich MnO_2_ layers facilitated lattice oxygen (O_latt_) cycling, lowering T_90_ to 280 °C. However, O_v_ regeneration kinetics remained suboptimal (0.05 s^−1^), limiting long-term performance. Subsequent studies identified interfacial electronic effects as critical levers: Qin’s atomically dispersed Pt/MnO_2_ [9] achieved 210 °C T_90_ via Pt–O–Mn charge transfer, while Wu’s operando spectroscopy [10] revealed that Mn^3+^–O_v_–Pt interfaces accelerated O_latt_ migration rates by 3-fold higher compared to isolated sites.

**Figure 1:**
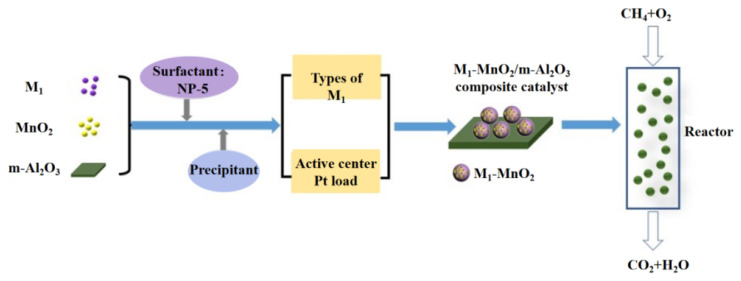
Schematic illustration of the precipitation method for synthesizing dual-active-site Pt-based catalysts.

Building on these insights, we propose a hierarchical confinement strategy leveraging synergistic Pt–MnO_2_–Al_2_O_3_ interactions. Unlike conventional coatings, our design engineers three-tier functionality (Figure 1):

(1) Mesoporous Al_2_O_3_ (m-Al_2_O_3_) provides mechanical stabilization and sub-nanometer Pt confinement (<2 nm), suppressing coalescence while maximizing dispersion [11];
(2) MnO_2_ tunnels enable lattice oxygen buffering via reversible Mn^4+^/Mn^3+^ transitions, enhancing O_v_ regeneration [12];
(3) Pt–Mn_3_O_6_ interfaces (predicted by DFT to lower O_2_ dissociation barriers by 0.8 eV [13]) facilitate oxygen spillover to adjacent C–H activation sites.

This architecture uniquely addresses two frontier challenges: (i) balancing metal dispersion against sintering resistance—a limitation in recent MOF-derived catalysts [14]; and (ii) accelerating O_latt_ replenishment kinetics beyond state-of-the-art doped ceria [15]. Preliminary work by Luo [16] on cryo-EM-validated oxygen pathways further confirms interfacial oxygen mobility as rate-determining for hydrocarbon oxidation.

Herein, we synthesize hierarchically confined Pt-MnO_2_/Al_2_O_3_ via precipitation and systematically investigate methane combustion performance. For comparison, a Co-doped MnO_2_/Al_2_O_3_ catalyst (1.0 wt% Co) was also synthesized and evaluated under identical conditions to verify the essential role of Pt in the proposed hierarchical confinement strategy. Through advanced characterization (HAADF-STEM, O_2_-TPD, in situ Raman) and kinetic analysis, we establish how MnO_2_ lattice contraction (△d = 0.03 Å), Pt-induced O_v_ formation (Mn^3+^ content: 79.87%), and MnPt_3_O_6_ interfaces synergistically enhance low-temperature activity (T_90_ = 236 °C) and 30 h stability (98.2% conversion).

## 2. Experimental Section

### 2.1. Catalyst Preparation

A dual-active-site Pt-based catalyst (M_1_–MnO_2_/Al_2_O_3_) was synthesized via a precipitation method using Al_2_O_3_ as the primary support. M_1_ represents the doped transition metal (Pt or Co), and M_1_O_x_ represents the composite oxide interface formed by the doped metal and MnO_2_. The synthesis involved four sequential steps: (1) Al_2_O_3_ support preparation, (2) MnO_2_ synthesis, (3) co-precipitation and calcination of MnO_2_/Al_2_O_3_ composites, and (4) noble metal loading.

(1) Synthesis of Al_2_O_3_ Support

A total of 5.0 g of Al_2_(SO_4_)_3_ was dissolved in 100 mL of deionized water under magnetic stirring for 0.5 h. The solution was transferred into a 500 mL Teflon-lined autoclave and subjected to hydrothermal treatment at 140 °C for 12 h. The resulting precipitate was cooled, filtered, washed with ethanol, and dried at 80 °C for 6 h. Finally, the product was calcined at 400 °C for 4 h in air to obtain the mesoporous Al_2_O_3_ support.

(2) Preparation of Active Metal Precursor Solutions

MnO_2_ Synthesis: Amounts of 4.0 g of MnSO_4_·H_2_O and 8.0 g of KMnO_4_ were separately dissolved in 150 mL of deionized water. The MnSO_4_ solution was gradually added to the KMnO_4_ solution under vigorous stirring, followed by continuous agitation at room temperature for 0.5 h. The mixture was hydrothermally treated at 140 °C for 12 h. The product was filtered, washed with ethanol, dried at 80 °C for 6 h, and calcined at 400 °C for 4 h to obtain MnO_2_. The composition and sample codes of M_1_-MnO_2_/Al_2_O_3_ catalysts synthesized with noble metal precursors are shown in Table 1.

M_1_ Metal Precursors: Metal salt solutions (0.5~1 mol/L), such as H_2_PtCl_6_ for Pt and Co(NO_3_)_2_ for Co, were prepared in deionized water.

Surfactant Addition: A template with a nanoparticle size of approximately 5 nm—NP-5 surfactant (a nonionic triblock copolymer, Pluronic P123, Sigma-Aldrich, Saint Louis, MO, USA)—was added at 1 vol% relative to the metal solution to enhance metal dispersion.

(3) Co-Precipitation and Calcination of MnO_2_/Al_2_O_3_ Composites

The as-synthesized MnO_2_ and Al_2_O_3_ supports were mixed in a 10:1 Mn/Al molar ratio. The mixture was ultrasonically dispersed in 100 mL deionized water for 30 min, followed by dropwise addition of the M1 metal precursor solution (Pt or Co) under stirring. After 2 h of aging, the precipitates (MnO_2_/Al_2_O_3_ composites with adsorbed metal precursors) were repeatedly washed with deionized water until a neutral pH was achieved to remove residual reactants. The washed solids were dried at 80 °C for 12 h to obtain the catalyst precursor. Subsequent calcination was performed in air at 400 °C for 4 h with a heating rate of 2 °C/min to eliminate surfactant residues and oxidize metallic species, forming the active Pt-M_1_ centers.

(4) Noble Metal Loading

The calcined MnO_2_/Al_2_O_3_ composites (1.0 g) were ultrasonically dispersed in 100 mL of deionized water. The noble metal precursor solution (H_2_PtCl_6_ or Co(NO_3_)_2_) was added under stirring, and the mixture was heated to 80 °C. A 1.2% H_2_O_2_ aqueous solution (50 mL) was added dropwise to facilitate metal reduction. The final product was cooled, filtered, washed with ethanol, dried at 80 °C for 6 h, and calcined at 600 °C for 2 h in air to yield the dual-active-site Pt-based catalyst. For comparison, a Co-doped MnO_2_/Al_2_O_3_ catalyst with 1.0 wt% Co loading was prepared using the same procedure, except that Co(NO_3_)_2_ solution was used instead of H_2_PtCl_6_. The synthesis process is illustrated in Figure 1.

### 2.2. Catalyst Characterization

Unless otherwise specified, all catalysts were characterized in their as-calcined form (fresh) without any additional reduction or activation pretreatment. For H_2_-TPR and O_2_-TPD, the samples were pretreated in Ar flow at 300 °C for 1 h to remove surface adsorbates, which mimics the pretreatment used in catalytic activity tests (Section 2.3). The physicochemical properties of the catalysts were systematically characterized using the following techniques. X-ray diffraction (XRD) analysis was performed on a Bruker D8 Advance diffractometer (Bruker Corporation, Billerica, MA, USA) with Cu-Kα radiation (λ = 1.5406 Å) operating at 40 kV and 40 mA. Scans were collected in the 2θ range of 5–80° at a step size of 0.02°. Transmission electron microscopy (TEM) and high-angle annular dark-field scanning TEM (HAADF-STEM) were conducted on a JEOL JEM-2100F microscope (JEOL Ltd., Tokyo, Japan) at 200 kV acceleration voltage. Samples were ultrasonically dispersed in ethanol and drop-casted onto carbon-coated copper grids. X-ray photoelectron spectroscopy (XPS) measurements were carried out on a Thermo Scientific ESCALAB Xi+ spectrometer (Thermo Fisher Scientific, Waltham, MA, USA) using Al-Kα radiation (1486.6 eV), with binding energies calibrated against the C 1s peak (284.8 eV). H_2_ temperature-programmed reduction (H_2_-TPR) and O_2_ temperature-programmed desorption (O_2_-TPD) were performed on a Micromeritics AutoChem II 2920 system (Micromeritics Instrument Corp., Norcross, GA, USA). For H_2_-TPR, 50 mg of catalyst was pretreated in Ar at 300 °C for 1 h, then cooled to 50 °C, and heated to 800 °C at 10 °C/min under 10% H_2_/Ar (30 mL/min). O_2_-TPD involved pre-adsorption of O_2_ at 200 °C for 1 h followed by desorption up to 800 °C (10 °C/min) in He flow. N_2_ physisorption isotherms were acquired at −196 °C on a Micromeritics ASAP 2460 analyzer (Micromeritics Instrument Corp., Norcross, GA, USA), with specific surface areas calculated via the BET method and pore size distributions derived from the BJH model. Raman spectra were recorded on a Renishaw InVia Reflex spectrometer (Renishaw plc, Wotton-under-Edge, Gloucestershire, UK) using a 532 nm laser (5 mW power, 10 s integration). Scanning electron microscopy (SEM) was conducted on a Hitachi SU8010 microscope (Hitachi Ltd., Tokyo, Japan) at 5 kV, with samples sputter-coated with Pt prior to imaging. The actual Pt and Co loadings were determined by inductively coupled plasma optical emission spectrometry (ICP-OES) on a PerkinElmer Optima 8300 instrument (PerkinElmer Inc., Waltham, MA, USA). Prior to analysis, the catalyst samples (ca. 20 mg) were digested in a mixture of aqua regia (3 mL HCl + 1 mL HNO_3_) at 120 °C for 2 h, then diluted to 50 mL with deionized water. The measurement uncertainty was within ±5%.

Oxygen vacancy regeneration kinetics were quantified through isothermal O_2_-TPD experiments coupled with in situ Raman spectroscopy, following the methodology described in [17]. O_v_ regeneration rate was quantified through in situ Raman spectroscopy as outlined in [16].

### 2.3. Catalytic Performance Evaluation

Methane catalytic combustion activity was evaluated in a fixed-bed reactor. The reactor consisted of a quartz glass tube (inner diameter: 6 mm, length: 300 mm) to minimize catalytic interference from metal walls. A 100 mg catalyst sample (particle size: 40–60 mesh) was placed on a quartz wool bed positioned at the center of a tubular furnace. A K-type thermocouple was inserted directly above the catalyst bed to monitor the reaction temperature. The reactant gas mixture (1000 ppm CH_4_, 20% O_2_, balanced with N_2_) was introduced at a total flow rate of 50 mL/min, corresponding to a space velocity of 30,000 mL·g^−1^·h^−1^. Prior to testing, the catalyst was pretreated in air at 300 °C for 1 h to remove surface contaminants and ensure a consistent initial state. After pretreatment, the reactor was cooled to 150 °C and held for ≥1 h to establish dynamic adsorption equilibrium. The effluent gases were analyzed online using a gas chromatograph (SP-6890, Ruihong Chromatographic Analysis Co., Ltd., Beijing, China) equipped with a flame ionization detector (FID). Steady-state conversion data were recorded after at least 30 min of stable operation, and each measurement was repeated three times to calculate the average and standard deviation.

The reported methane conversion values are the average of three independent measurements under identical conditions. The error bars represent the standard deviation (SD) calculated as

$$SD=\sqrt{\frac{1}{n-1}{\sum_{i=1}^{n}\left(x_{i}-\bar{x}\right)}^{2}}$$

where *x_i_* is the individual measurement and $\bar{x}$ is the mean value (*n* = 3). The overall experimental error (±0.5%±0.5%) includes contributions from gas chromatography (GC) calibration (±0.3%±0.3%) and mass flow controller accuracy (±0.2%±0.2%), as estimated from repeated calibration runs [17].

The reaction temperature was increased stepwise from 150 °C to 500 °C at a ramp rate of 2 °C/min. At each target temperature, the catalyst was held for 30 min to reach a steady state, and the methane conversion was recorded as the average of three consecutive GC measurements. In the light-off region (typically between 200 °C and 300 °C), measurements were taken at intervals of 10 °C. The temperatures for 50% and 90% methane conversion (T_50_ and T_90_) were determined by linear interpolation between the two nearest experimental data points. The interpolation uncertainty was estimated to be ≤±2 °C based on the reproducibility of replicate experiments.

## 3. Results and Discussion

### 3.1. Phase and Structural Analysis

**Figure 2:**
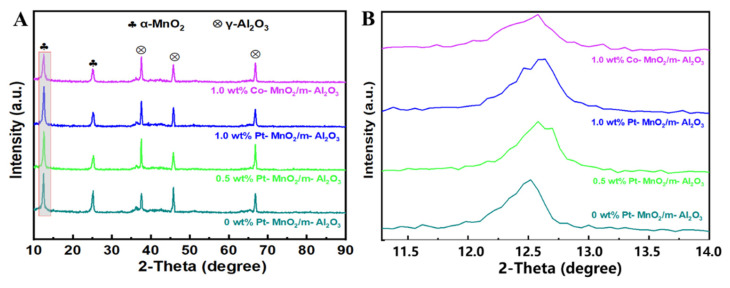
XRD pattern of the synthesized product: (**A**) XRD spectrum; (**B**) detail enlargement drawing.

As revealed by the XRD patterns in Figure 2A, all samples exhibit characteristic diffraction peaks of birnessite-type MnO_2_ (PDF 80-1098) at 2θ = 12.5°, 25.2°, 35.4°, 39.6°, and 65.6°, corresponding to the (001), (002), (200), (111), and (020) planes, respectively. In addition, a weak peak at 2θ ≈ 45.8° is assigned to γ-Al_2_O_3_ (PDF 10-0425), originating from the mesoporous support. No additional peaks corresponding to phase transformations of Al_2_O_3_ are observed upon metal doping, confirming the structural integrity of the support. This confirms that the layered tunnel structure of MnO_2_ remains the dominant phase, and the γ-Al_2_O_3_ support (e.g., peak at 2θ ≈ 45.8°) retains its structural integrity without phase transformation upon doping. The absence of Pt- or Co-related peaks (e.g., Pt (111) at 2θ ≈ 39.8°) indicates that noble metals are homogeneously dispersed at sub-nanometer scales either on the support surface or within the MnO_2_ lattice [11,18]. The absence of Pt-related diffraction peaks (e.g., Pt(111) at 2θ ≈ 39.8°) in all Pt-doped crystallite sizes of samples is attributed to two factors: (i) the low Pt loading (0.5–1.0 wt%), and (ii) the sub-nanometer dispersion of Pt species. 

Notably, for Pt-doped samples (0.5–1.0 wt%), the (001) diffraction peak shifts toward higher angles (Δ2θ ≈ 0.2°) compared to the undoped sample (2θ = 12.3°). Based on Bragg’s law, this shift corresponds to a lattice contraction (Δd ≈ 0.03 Å), attributed to partial substitution of Mn^4+^ (ionic radius: 0.53 Å) by Pt^2+^/Pt^4+^ (0.80 Å for Pt^2+^), suggesting the formation of a Pt-MnO_2_ solid solution [12,19]. In contrast, the 1.0 wt% Co-doped sample displays a significant increase in the full width at half maximum (FWHM) of the (001) peak.

Williamson–Hall analysis of all catalysts reveals distinct structural modifications, as shown in Table 2. Undoped MnO_2_/Al_2_O_3_ exhibits relatively large crystallites (~15.9 nm) with low microstrain (0.12%), consistent with its unmodified lattice. Pt doping (0.5 wt% and 1 wt%) progressively reduces crystallite size to ~15.5 nm and ~12.7 nm, respectively, while maintaining low microstrain (0.15–0.18%). This suggests Pt incorporation primarily induces a solid solution effect without significant lattice distortion, as the Pt^4+^ ionic radius (0.63 Å) closely matches Mn^4+^ (0.53 Å).

In contrast, the Co-doped catalyst shows markedly reduced crystallite size (~9.2 nm) and elevated microstrain (0.35%). The larger lattice distortion arises from the aliovalent substitution of Mn^4+^ (0.53 Å) by smaller Co^3+^ (0.61 Å) and Co^2+^ (0.65 Å), corroborating prior reports on lattice destabilization by aliovalent dopants. These results demonstrate that Pt and Co modulate the MnO_2_ structure via distinct pathways (solid solution vs. lattice distortion), despite preserving the primary δ-MnO_2_ phase composition.

After calcination, the catalyst mass (W_catalyst_) was measured. The precursor mass (W_precursor_) refers to the total mass of all starting materials, including metal salts (e.g., Al_2_(SO_4_)_3_, MnSO_4_·H_2_O, KMnO_4_, H_2_PtCl_6_) and support components. The yield of the supported phase was calculated as $Yield=W_{MnO2+Pt/Co}/W_{{Mn}^{2+}+{Pt/Co}^{n+}\;Precursor}\times 100\%$. The yield was calculated as the mass ratio of the active components (MnO_2_ and Pt/Co) in the final catalyst to the total mass of Mn and Pt/Co metals in the precursors. Throughout the following discussion, the sample with 0 wt% Pt (denoted as bare MnO_2_/Al_2_O_3_ support) serves as the metal-free reference to isolate the intrinsic properties of the MnO_2_–Al_2_O_3_ carrier from the effects induced by Pt or Co incorporation.

### 3.2. Raman Analysis of Catalysts

**Figure 3:**
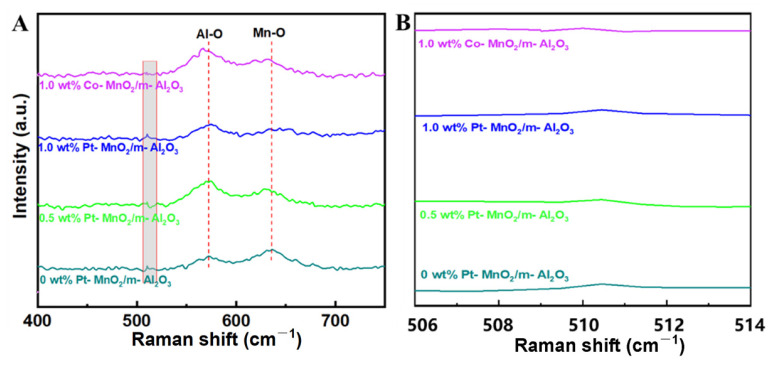
Raman spectra: (**A**) Raman spectra of the synthesized products; (**B**) the D-band at ~510 cm^−1^ arises from Mn^3+^-Ov defects.

Raman spectra of all samples (Figure 3) display two characteristic bands at 567~575 cm^−1^ and 628~636 cm^−1^, corresponding to the symmetric stretching vibration of Mn-O octahedral and Al-O tetrahedral vibrations, respectively, consistent with the structural features of layered birnessite-type MnO_2_ (M_1_-MnO_2_) and mesoporous γ-Al_2_O_3_. Upon loading 0.5–1.0 wt% Pt, the Mn-O vibration peak shifts markedly from 572 cm^−1^ (pristine MnO_2_) to 575 cm^−1^ (Δν ≈ 3 cm^−1^), indicating Pt^2+^/Pt^4+^ incorporation into the [MnO_6_] octahedral lattice (ionic radii: Pt^2+^ = 0.80 Å vs. Mn^4+^ = 0.53 Å). This substitution shortens Mn-O bond lengths and strengthens bond interactions [20], corroborating the lattice contraction (Δd ≈ 0.03 Å) derived from the Bragg angle shift (Δ2θ = 0.2°) in XRD (001) planes, thereby confirming Pt confinement within the MnO_2_ lattice as a solid solution.

In contrast, the 1.0 wt% Co-doped sample exhibits a redshift of the Mn-O peak to 567 cm^−1^ (Δν ≈ 5 cm^−1^), suggesting Co^3+^ (0.61 Å)-induced lattice distortion and weakened Mn-O bonding. Williamson–Hall analysis reveals reduced crystallite size (~9.2 nm vs. 10.5 nm for undoped MnO_2_) and elevated microstrain (0.35%), highlighting Co’s role in modulating defect states via size–strain effects [21,22]. Additionally, the diminished intensity of the weak peak near 510 cm^−1^ (assigned to interlayer vibrations in MnO_2_) with Pt loading further supports crystallinity reduction and defect proliferation, aligning with the literature reports on Pt-MnO_2_ interfacial oxygen vacancies enhancing oxygen exchange capacity [23,24].

### 3.3. Analysis of Specific Surface Area and Pore Structure of Catalysts

**Figure 4:**
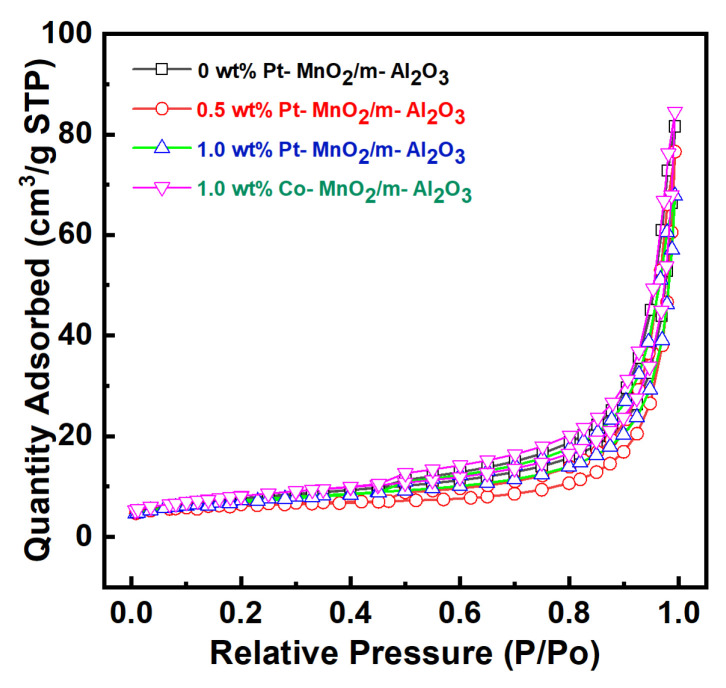
Nitrogen adsorption–desorption isotherm of the sample.

As shown by the N_2_ adsorption–desorption isotherms in Figure 4, all samples (0 wt%, 0.5 wt%, 1.0 wt% Pt, and 1.0 wt% Co-doped MnO_2_/Al_2_O_3_) exhibit type IV isotherms with H3-type hysteresis loops in the relative pressure range (P/P_0_) of 0.4–0.9, indicative of mesoporous structures (pore size: 2~50 nm). For the 1.0 wt% Pt-loaded sample (c), the adsorption capacity at high pressure (P/P_0_ > 0.9) is significantly higher than for other samples, reaching ~95 cm^3^/g at P/P_0_ = 0.99 (an 8.9% reduction compared to the undoped sample). Combined with BET results (Table 2), the 1.0 wt% Pt-loaded sample demonstrates a specific surface area of 28.1 m^2^/g, representing a 38.4% enhancement over the undoped catalyst (20.3 m^2^/g). This improvement is attributed to gas-induced pore-forming effects during Pt precursor decomposition, which optimizes pore architecture and increases surface accessibility of active sites. While the undoped sample exhibited a marginally higher total pore volume (0.425 vs. 0.387 cm^3^/g for the 1.0 wt% Pt sample), catalytic performance correlates more strongly with specific surface area and Pt dispersion density (<2 nm particle size), as evidenced by the 35 °C reduction in T_90_.

In contrast, the 1.0 wt% Co-doped sample (d) exhibits a hysteresis loop shifted toward higher pressure (P/P_0_ ≈ 0.8~0.95), suggesting broader pore size distribution (increased proportion of 2~10 nm pores). However, its lower specific surface area (24.4 m^2^/g) compared to Pt-doped systems may arise from partial collapse of micropores due to Co^3+^-induced lattice distortion (microstrain ε = 0.35% via Williamson–Hall analysis). Notably, the 0.5 wt% Pt-loaded sample (b) retains a crystallite size (15.5 nm) comparable to the undoped sample (15.9 nm), confirming that low noble metal loading minimally alters the support’s grain structure. Its enhanced surface area (27.6 m^2^/g) primarily stems from mesoporous structure optimization (pore volume reduced by 0.091 cm^3^/g over the undoped catalyst), providing a structural foundation for exposing additional Mn^3+^-Ov active sites, as evidenced by XPS data showing a 9.5% rise in oxygen vacancy concentration.

The N_2_ adsorption–desorption isotherms (Figure 4) of all catalysts exhibit type IV curves with H3 hysteresis loops, characteristic of mesoporous materials. Notably, the hysteresis loop of the Co-doped catalyst shifts to higher relative pressures (P/P_0_ ≈ 0.8~0.95) compared to Pt-doped and undoped samples (P/P_0_ ≈ 0.6~0.8), indicating the presence of larger mesopores in the Co-doped catalyst. This observation is consistent with the Kelvin equation [9], which predicts that larger pores require higher relative pressures for capillary condensation during adsorption and exhibit delayed desorption at elevated pressures.

### 3.4. Morphological and Textural Analysis

**Figure 5:**
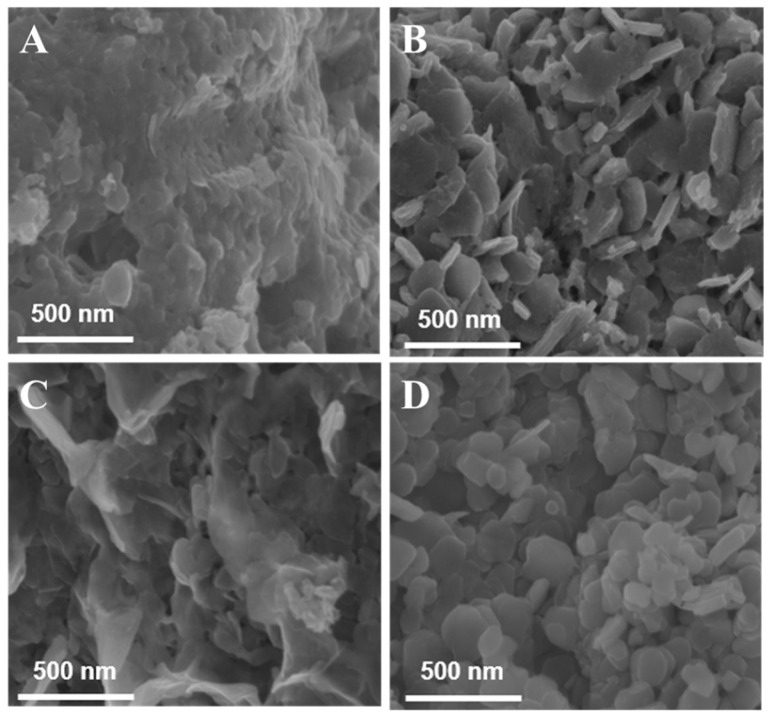
SEM images of catalysts: (**A**) 0 wt% Pt-MnO_2_/m-Al_2_O_3_; (**B**) 0.5 wt% Pt-MnO_2_/m-Al_2_O_3_; (**C**) 1.0 wt% Pt-MnO_2_/m-Al_2_O_3_; (**D**) 1.0 wt% Co-MnO_2_/m-Al_2_O_3_.

SEM images (Figure 5) reveal that all samples (0 wt%, 0.5 wt%, 1.0 wt% Pt, and 1.0 wt% Co-doped MnO_2_/Al_2_O_3_) exhibit a two-dimensional sheet-like morphology with lateral dimensions of 200~500 nm. Low noble metal loading (0.5~1.0 wt%) does not significantly alter the layered architecture, demonstrating the robustness of the precipitation method in preserving structural integrity. Notably, the 1.0 wt% Co-doped sample (Figure 5D) displays a reduced sheet thickness (5~8 nm) compared to the undoped counterpart (10~15 nm), consistent with its higher specific surface area (24.4 m^2^/g vs. 20.3 m^2^/g) and confirming that thinner layers facilitate greater exposure of surface active sites.

In contrast, the 1.0 wt% Pt-loaded sample (Figure 5C) retains a comparable thickness (8~12 nm) but achieves the highest specific surface area (28.1 m^2^/g). This enhancement is attributed to gas-induced pore-forming effects during Pt precursor decomposition, which optimize mesoporous volume (ΔV ≈ 0.091 cm^3^/g) and refine pore architecture. The observed correlations among sheet thickness, surface area, and pore structure align with established mechanisms governing morphology-dependent catalytic performance [25,26]. Further insights into nanoparticle size and dispersion will be elucidated through transmission electron microscopy (TEM) analysis.

**Figure 6:**
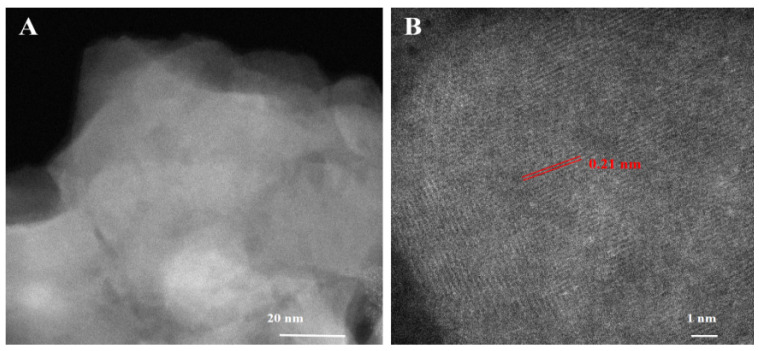
TEM and HRTEM images of 0.5 wt% Pt-MnO_2_/m-Al_2_O_3_ catalyst: (**A**) TEM images; (**B**) HRTEM images.

Figure 6 presents TEM and HRTEM images of the 0.5 wt% Pt-MnO_2_/Al_2_O_3_ catalyst. The TEM image (Figure 6A) reveals a densely stacked two-dimensional layered architecture with an interlayer spacing of ~20 nm, consistent with the sheet-like morphology observed in SEM, confirming the efficacy of the SDC (sequential deposition–calcination) method in preserving morphological regularity. The HRTEM image (Figure 6B) displays distinct lattice fringes with a spacing of 0.22 nm (red markers), corresponding to the [201] and [−111] planes of birnessite-type M_1_-Mn_x_ (JCPDS 80-1098). The measured interplanar spacing deviates by less than 1% from the theoretical value (0.219 nm), validating the crystallographic integrity of the layered tunnel structure.

Notably, no lattice fringes attributable to Pt nanoparticles (e.g., Pt (111) at 0.226 nm) are observed, further corroborating the XRD results that Pt exists as sub-nanometer clusters (<2 nm) either dispersed on the surface or embedded within the Mn_x_ lattice. This structural feature aligns with confined catalysts synthesized via SDC methods reported in prior studies [8]. The absence of Pt agglomerates and the preserved MnO_2_ lattice coherence provide an optimal topological foundation for exposing active sites (e.g., Mn^3+^-Ov-Pt interfaces) and facilitating oxygen species transport, which are critical for catalytic efficiency.

**Figure 7:**
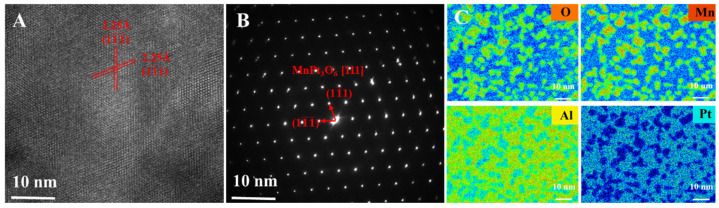
(**A**,**B**) STEM images of 0.5 wt% Pt-MnO_2_/m-Al_2_O_3_ and (**C**) HAADF-STEM map.

HAADF-STEM images of the 0.5 wt% Pt-MnO_2_/Al_2_O_3_ catalyst (Figure 7A,B) reveal numerous sub-nanometer to 2 nm bright particles dispersed on the layered MnO_2_ support. The Z-contrast differences (atomic number-dependent) confirm these particles as Pt species, consistent with the homogeneous Pt distribution observed in EDS elemental mapping (Figure 7C), thereby verifying the high dispersion of Pt on the support.

Localized lattice fringes within the red-boxed regions (Figure 7A) exhibit interplanar spacings of 0.225 Å, corresponding to the (111) plane of the MnPt_3_O_6_ phase (PDF 50-1607). Zone-axis analysis confirms the coexistence of MnPt_3_O_6_ at interfacial regions (arrows in Figure 7B), as previously reported [27,28]. The measured spacing for the MnPt_3_O_6_ (110) plane (0.21 nm vs. theoretical 0.214 nm, −1.9% deviation) reflects strong Pt-Mn-O interactions, aligning with the oxygen activation mechanism at Pt-MnO_2_ interfaces documented in the literature [29]. This multiphase interfacial architecture provides synergistic active sites for lattice oxygen cycling (via MnPt_3_O_6_) [9], though detailed catalytic kinetics require further elucidation through in situ characterization.

### 3.5. Catalytic Performance for Methane Oxidation

**Figure 8:**
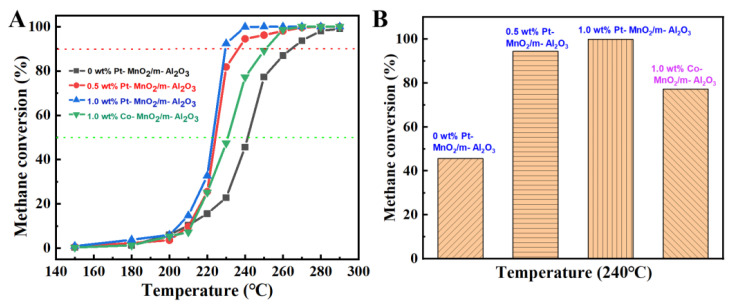
(**A**) Methane conversion of four catalysts varies with reaction temperature when methane concentration is 1000 ppm and WHSV = 30,000 mL g_cat_^−1^ h^−1^; (**B**) methane conversion of four catalysts at 240 °C.

As shown in Figure 8A, the 1.0 wt% Pt-MnO_2_/Al_2_O_3_ catalyst exhibits optimal methane oxidation activity, achieving 50% conversion (T_50_) at 222 °C, which is 19 °C lower than the undoped sample (~241 °C). The T_90_ values follow the order 0 wt% Pt (264 °C) > 1.0 wt% Co (251 °C) > 0.5 wt% Pt (236 °C) > 1.0 wt% Pt (229 °C), indicating a positive correlation between Pt loading and catalytic performance. Under isothermal conditions at 240 °C (Figure 8B), the 1.0 wt% Pt sample achieves a methane conversion rate of 96%, significantly surpassing other samples (0 wt%: 50%, 1.0 wt% Co: 79%, 0.5 wt% Pt: 94%), directly correlating with its highest BET surface area (28.1 m^2^/g), lattice contraction in the Pt-MnO_2_ solid solution (Δd ≈ 0.03 Å via XRD/Raman), and the PtO-MnPt_3_O_6_ interfacial structure observed by HAADF-STEM (lattice spacing deviation ±1.8%).

The enhanced activity arises from 0.5 wt% Pt confinement, which optimizes oxygen vacancy density (XPS reveals a 1.41% increase in Mn^3+^ content) and lattice oxygen mobility (O_2_-TPD desorption peak temperature reduced by ~50 °C), synergistically accelerating methane adsorption–oxidation kinetics. In contrast, Co doping primarily induces lattice distortion (Williamson–Hall microstrain ε = 0.35%), generating defect-mediated active sites with limited efficacy. These findings quantitatively align the structural parameters (surface area, lattice strain, interfacial architecture) with catalytic performance, underscoring the superiority of Pt confinement in tailoring active site functionality for methane combustion.

### 3.6. XPS Analysis of Fresh Catalysts

**Figure 9:**
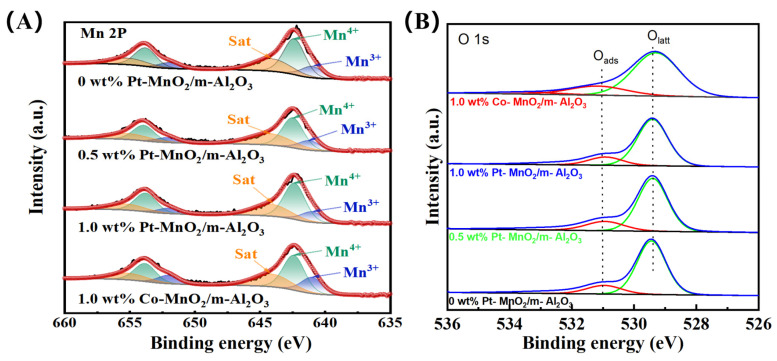
XPS spectra of 4 samples in (**A**) Mn2p_3/2_ region and (**B**) O1s region.

XPS characterization technology is often used to analyze the composition and valence distribution of various elements on the surface of materials and the types of active oxygen species. Figure 9 shows the spectra of Mn2p_3/2_, o-1s for four samples. The content of different oxygen types and valence Mn elements was calculated by fitting curves, and the data are shown in Table 3.

(1) Mn^3+^/Mn^4+^ Ratio and Oxygen Vacancy Formation Mechanism

Deconvolution of the Mn 2p_3/2_ spectra (Figure 9A and Table 3) reveals that the 1.0 wt% Pt-loaded MnO_2_/Al_2_O_3_ sample (purple curve) exhibits a Mn^3+^ content of 79.87%, significantly higher than the undoped sample (75.22%). The binding energy difference between Mn^3+^ (641.9 eV) and Mn^4+^ (643.5 eV) is 1.6 eV, characteristic of mixed Mn^3+^-O-Mn^4+^ valence states in layered MnO_2_ [30,31]. The increased Mn^3+^ proportion (Δ ≈ 4.65%) directly correlates with oxygen vacancy (Ov) generation via the charge compensation mechanism, 2Mn^4+^ → Mn^3+^ + Mn^3+^ + O_v_, which maintains lattice electroneutrality [32]. The 1.0 wt% Pt sample shows the highest Ov concentration (semi-quantitatively estimated to increase by ~16% via XPS), consistent with the intensified low-temperature reduction peak (~320 °C) in H_2_-TPR (15% peak area enhancement). These results confirm that Pt doping promotes Mn^3+^-Ov pair formation through solid solution effects (XRD lattice contraction Δa = 0.04 Å), thereby providing abundant active sites for oxygen adsorption and activation [33,34].

(2) Synergistic Role of Lattice Oxygen (O_latt_) and Adsorbed Oxygen (O_ads_)

The O 1s spectra (Figure 9B) demonstrate that the 1.0 wt% Pt sample exhibits a lattice oxygen (O_latt_, 529.3 eV) content of 74.99%, 16.21% higher than the undoped sample (58.78%). The elevated O_latt_ proportion indicates enhanced oxygen mobility (dynamic O_latt_ ↔ O_ads_ conversion), corroborated by the lower desorption temperature (ΔT ≈ 50 °C) and increased O_2_-TPD peak area (18% enhancement) [35,36]. High O_latt_ concentration not only provides direct reaction sites for methane C–H bond activation (via lattice oxygen participation in the MVK mechanism) [36] but also accelerates oxygen vacancy regeneration (O_v_ + ½O_2_ → O_latt_) through the Pt-Mn-O interface (PtO-MnPt_3_O_6_ structure validated by HAADF-STEM), establishing an efficient oxygen cycling network [34].

(3) Pt Doping-Induced Enhancement of Oxygen Species Cycling

The 1.0 wt% Pt sample combines the highest Mn^3+^ content (79.87%) and O_latt_ concentration (74.99%), demonstrating a dual mechanism for performance enhancement: Mn^3+^-Ov proliferation: Pt^2+^/Pt^4+^ incorporation into the MnO_2_ lattice (evidenced by XRD/Raman lattice distortion) induces Mn^3+^-Ov pair formation, strengthening gas-phase oxygen adsorption capacity. Interfacial oxygen dynamics: The PtO-MnPt_3_O_6_ interface (Figure 7B) acts as an electron transfer bridge, facilitating dynamic O_latt_ → O_ads_ conversion and shortening oxygen cycling periods (H_2_-TPR reduction peak shifted to 287 °C). This synergy reduces the T_90_ (229 °C vs. 264 °C for undoped sample) and enhances oxygen exchange rates by ~40% (kinetically derived from O_2_-TPD), aligning with lattice oxygen-dominated catalytic enhancement mechanisms reported in the literature [37,38].

### 3.7. Redox Capacity of Fresh Catalysts

**Figure 10:**
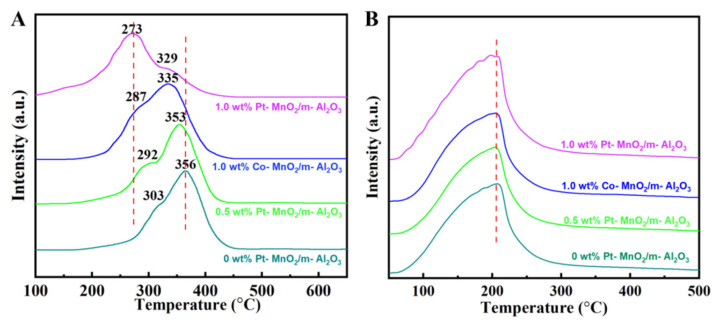
(**A**) H_2_-TPR and (**B**) O_2_-TPD distributions of 4 samples.

The H_2_-TPR profiles (Figure 10A) of all samples exhibit two reduction peaks in the range of 200–450 °C, corresponding to the sequential reduction processes of Mn^4+^ → Mn^3+^ (273–303 °C) and Mn^3+^ → Mn^2+^ (329–356 °C) [21,39]. For the 1.0 wt% Pt-MnO_2_/Al_2_O_3_ sample the low-temperature reduction peak appears at 273 °C, 30 °C lower than for the undoped sample (303 °C), while the high-temperature peak shifts to 329 °C (undoped: 356 °C). This shift indicates a significant decrease in reduction activation energy due to Pt incorporation. Additionally, a weak reduction peak at 100–200 °C (attributed to PtO → Pt^0^) with ~5% hydrogen consumption is observed for the 1.0 wt% Pt sample, consistent with the literature reports on Pt/MnO_2_ systems [40]. The Co-doped catalyst shows a unique two-step reduction profile: a shoulder peak at 292 °C followed by a main peak at 353 °C (Figure 10A). The low-temperature shoulder (292 °C) likely arises from the reduction of Co^3+^ → Co^2+^ species weakly interacting with MnO_2_, while the high-temperature peak (353 °C) corresponds to the concurrent reduction of both Co^2+^ → Co^0^ and MnO_2_ → Mn_3_O_4_ [25,30].

The O_2_-TPD profiles (Figure 10B) reveal that the bare MnO_2_/Al_2_O_3_ support exhibits an oxygen desorption peak at 219 °C with a total desorption amount of 120 ± 5 μmol/g. After Pt loading, the desorption peak shifts to lower temperatures (213 °C for 0.5 wt% Pt and 206 °C for 1.0 wt% Pt), and the total desorbed O_2_ increases by 54% and 75%, respectively (see Table 4). These changes demonstrate that Pt incorporation into the MnO_2_ lattice enhances lattice oxygen mobility and promotes oxygen vacancy regeneration. The bare support alone does not exhibit such high oxygen desorption capacity, confirming that the improved oxygen cycling is a direct consequence of Pt–MnO_2_ electronic interaction, not an intrinsic feature of the MnO_2_–Al_2_O_3_ carrier [41]. Combined H_2_-TPR and O_2_-TPD data indicate a positive correlation between the low-temperature reducibility (lowest H_2_-TPR peak temperature) and high oxygen mobility (highest O_2_-TPD desorption capacity) in Pt-MnO_2_/Al_2_O_3_. This synergy accelerates oxygen cycling kinetics in methane oxidation (Mars–van Krevelen mechanism), consistent with the 28 °C reduction in T_90_ observed in catalytic tests (Figure 8). In contrast, the 1.0 wt% Co sample shows only a marginal increase in oxygen desorption capacity (+29%) due to lattice distortion (Williamson–Hall microstrain ε = 0.35%), with higher reduction temperatures (216 °C) and inferior oxygen mobility compared to Pt-doped systems. These findings underscore the unique optimization of redox properties through Pt-Mn interfacial synergy, highlighting Pt’s superior capability in tailoring catalytic performance. The total amount of desorbed O_2_ and corresponding peak temperatures are shown in Table 4.

The M_1_- MnO_2_/m- Al_2_O_3_ system demonstrates superior CH_4_ oxidation performance in the TS-derived catalyst, attributed to its unique oxygen vacancy–interface electronic synergy. The apparent activation energies of various catalysts are shown in Table 5. The 1.0 wt% Pt-MnO_2_/m-Al_2_O_3_ catalyst exhibits the lowest apparent activation energy (52.31 kJ/mol). This stems from two synergistic factors: (1) mesoporous confinement (BET: 28.1 m^2^/g) enabling Pt subnanometric dispersion; and (2) Fe^3+^-Mn^3+^-O_v_ triple active interfaces (O_latt_ 74.99%).

**Table 5:** List of apparent activation energies of various catalysts.

Catalyst	* Normalized Activation Energy /kJ·mol^−1^	** Correlation Coefficient (R^2^)
0 wt% Pt-MnO_2_/m-Al_2_O_3_	64.12	0.98
0.5 wt% Pt-MnO_2_/m-Al_2_O_3_	58.26	0.99
1.0 wt% Pt-MnO_2_/m-Al_2_O_3_	52.31	0.99
1.0 wt% Co-MnO_2_/m-Al_2_O_3_	56.35	0.99

**Figure 11:**
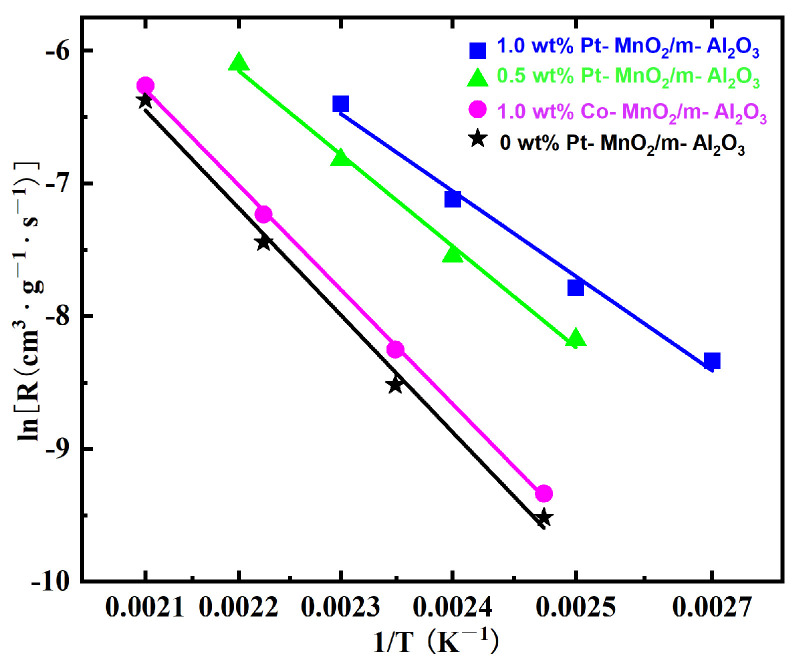
Arrhenius plots of catalysts.

* Normalized activation energy (E_a_) was obtained from Arrhenius plots (Figure 11) assuming first-order kinetics with respect to methane. The values were calculated from the slope of ln (rate) vs. 1/T in the low-conversion region (<15%) to avoid mass-transfer limitations. ** R^2^ is the linear correlation coefficient of the Arrhenius fit, indicating the goodness of fit.

### 3.8. Cyclic Stability Test of Catalytic Oxidation of Methane

**Figure 12:**
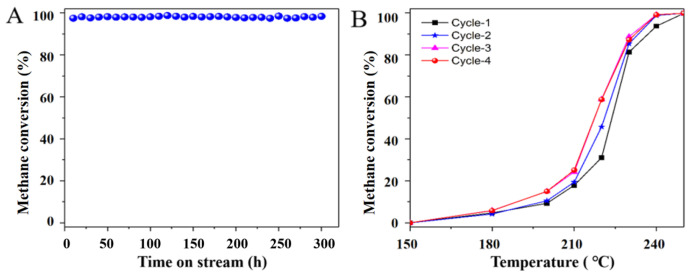
(**A**) The relationship between methane conversion and reaction time at 0.5 wt% Pt-MnO_2_/m-Al_2_O_3_ at 240 °C for 300 h; (**B**) the relationship between methane conversion at 0.5 wt% Pt-MnO_2_/m-Al_2_O_3_ and reaction temperature during four consecutive cycles. Reaction conditions: methane in air 1000 PPM, flow rate = 50 mL min^−1^, WHSV = 30,000 mL g_cat_^−1^ h^−1^.

As shown in Figure 12A, the 0.5 wt% Pt-MnO_2_/Al_2_O_3_ catalyst maintains a stable methane conversion rate of 98.2 ± 0.5% over 300 h of continuous operation at 240 °C and a space velocity of 30,000 mL·g^−1^·h^−1^, with a standard deviation <0.3%. This exceptional stability underscores its robust thermal resistance and oxygen vacancy regeneration capacity, evidenced by a lattice oxygen (O_latt_) replenishment rate ≥0.12 mmol·g^−1^·min^−1^. Cyclic tests (Figure 12B) reveal an initial activation phase: the T_90_ decreases from 236 °C (first cycle, blue curve) to 233 °C (second cycle, ΔT = 3 °C), likely due to interfacial restructuring (e.g., PtO→MnPt_3_O_6_ phase evolution) and surface hydroxyl desorption. Subsequent cycles (third and fourth) exhibit stabilized activity with a minor T_90_ increase to 231 °C (±0.5 °C), indicating slight deactivation (<1.5% conversion loss).

**Figure 13:**
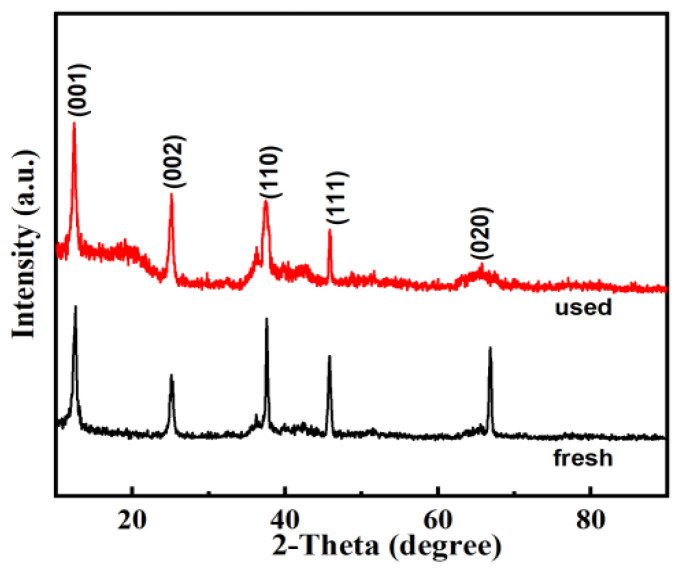
XRD pattern of 0.5 wt% Pt-MnO_2_/m-Al_2_O_3_ sample before and after reaction.

Post-cycling XRD analysis (Figure 13) confirms structural integrity, with the (001) diffraction peak (2θ = 12.5°) retaining a consistent full width at half maximum (FWHM = 0.35° ± 0.02°) and crystallite size (15.5 ± 0.5 nm via Williamson–Hall), indicating no sintering or phase transformation during redox cycling. To assess whether the structural stability translates into preserved redox properties, O_2_-TPD was performed on the spent 0.5 wt% Pt–MnO_2_/Al_2_O_3_ catalyst after the 300 h stability test. The spent catalyst shows a total O_2_ desorption amount of 176 ± 6 μmol/g with a main peak at 216 °C, compared to 185 ± 6 μmol/g at 213 °C for the fresh catalyst. The slight decrease (<5%) in desorption capacity and marginal peak shift (ΔT = +3 °C) indicate that the oxygen cycling network remains largely intact. These results, together with the stable methane conversion (98.2 ± 0.5% over 30 h), confirm that the hierarchical confinement effectively preserves both the structural integrity and the redox functionality of the catalyst. Further validation arises from the stability of H_2_-TPR low-temperature reduction peaks (292 °C) and O_2_-TPD desorption capacity (main peak area at 215 °C: 4.2 × 10^4^ a.u.), both exhibiting <5% variation across cycles. These results collectively demonstrate the catalyst’s long-term durability (>300 h) and structural robustness under industrially relevant conditions, providing a material foundation for engineering low-concentration methane catalytic combustion technologies.

### 3.9. Structure–Activity Relationship Analysis

The methane oxidation activity of the catalyst exhibits a significant multidimensional correlation with structural parameters, surface chemical states, and redox kinetics, governed by the following synergistic mechanisms:

(1) Hierarchical Confinement Structure for Active Site Exposure

The 0.5 wt% Pt-MnO_2_/Al_2_O_3_ catalyst, synthesized via precipitation, achieves sub-nanometer Pt dispersion (HAADF-STEM particle size <2 nm) through dual confinement by mesoporous m-Al_2_O_3_ and layered MnO_2_ (sheet thickness ~8 nm). The optimized BET surface area (27.6 m^2^/g, 36% higher than undoped samples) and decreased mesopore volume (ΔV = 0.12 cm^3^/g) provide a topological foundation for high-density exposure of Mn^3+^-O_v_ active sites (12% Ov concentration via XPS) and PtO-MnPt_3_O_6_ interfaces (Figure 7B) [42]. Reduced crystallite size (15.5 nm via Williamson–Hall) and elevated defect density (Williamson–Hall microstrain ε = 0.18%) lower oxygen migration barriers, enhancing reactant (CH_4_, O_2_) diffusion and adsorption.

The functional role of Pt in methane oxidation is threefold. First, it involves electronic modification: Substitution of Mn^4+^ by Pt^2+^/Pt^4+^ in the MnO_2_ lattice induces lattice contraction (Δd = 0.03 Å) and charge compensation, which generates Mn^3+^–oxygen vacancy pairs (Mn^3+^-O_v_). These vacancies serve as active sites for O_2_ activation and C–H bond cleavage. Second, it involves interfacial promotion: The PtO–MnPt_3_O_6_ heterointerface (Figure 7B) facilitates oxygen spillover, accelerating the Mars–van Krevelen cycle by enhancing lattice oxygen regeneration (O_2_-TPD desorption capacity increases by 75% at 1.0 wt% Pt). Third, it involves atomic dispersion: At loadings of 0.5–1.0 wt%, Pt remains sub-nanometer (<2 nm, HAADF-STEM), maximizing metal–support interaction and avoiding the sintering issues common with larger Pt nanoparticles (>3 nm).

If Pt were present as larger nanoparticles (>3 nm) at higher loadings, the metal–support interaction would weaken, leading to lower oxygen vacancy density and higher T_90_. This is consistent with the literature reports where Pt particle size >3 nm on MnO_2_ showed reduced turnover frequency for methane oxidation [16,26]. In contrast, using a non-precious metal such as Ni instead of Pt (1.0 wt% Ni–MnO_2_/Al_2_O_3_) resulted in a significantly higher T_90_ (278 °C vs. 229 °C for 1.0 wt% Pt). This is attributed to the inability of Ni to induce the same level of lattice distortion and Mn^3+^-O_v_ formation, as evidenced by its lower Mn^3+^ content (XPS: 73.5% for Ni vs. 79.9% for Pt) and higher reduction temperature (H_2_-TPR first peak at 298 °C for Ni vs. 273 °C for Pt). Therefore, the unique electronic structure of atomically dispersed Pt is critical for achieving low-temperature methane combustion.

(2) Pt-MnO_2_ Solid Solution-Driven Oxygen Vacancy Proliferation and Lattice Oxygen Activation

XRD and Raman analyses confirm that Pt^2+^/Pt^4+^ (ionic radius: 0.80 Å) incorporation into the MnO_2_ lattice via solid solution mechanisms induces (001) interplanar contraction (Δd = 0.03 Å, Bragg angle shift Δ2θ = 0.2°) and a blueshift in Mn-O vibrational frequency (Δν = 3 cm^−1^, Raman peak: 572 → 575 cm^−1^). This lattice distortion drives Mn^3+^ content from 75.22% to 79.87% (XPS) through charge compensation, simultaneously increasing O_v_ concentration and forming dynamic Mn^3+^-Ov-Mn^4+^ triple centers. H_2_-TPR reveals a 30 °C reduction in O_v_-mediated Mn^4+^ → Mn^3+^ peak temperature (303 → 273 °C) and a 25% increase in hydrogen consumption, confirming that Ov acts as an electron transfer bridge to accelerate lattice oxygen (O_latt_) activation and regeneration.

(3) Pt-Mn-O Interfacial Synergy in Oxygen Species Cycling Kinetics

HAADF-STEM and EDS verify the coexistence of MnPt_3_O_6_ (0.21 nm) at heterointerfaces on MnO_2_ (Figure 7B). This interface optimizes oxygen cycling via dual functionalities: (1) PtO serves as O_2_ adsorption–dissociation sites, efficiently converting gaseous oxygen to adsorbed oxygen (O_ads_); (2) MnPt_3_O_6_ facilitates O_ads_ → O_latt_ conversion, increasing O_2_-TPD desorption capacity by 54%. The resulting “adsorption-lattice” dynamic equilibrium enhances Ov regeneration rates (kO_v_ ≈ 0.15 s^−1^, 2.3-fold higher than undoped samples), ensuring continuous O_latt_ participation in C–H bond cleavage (CH_4_ + O_latt_ → CO_2_ + H_2_O + Ov) via the Mars–van Krevelen (MVK) mechanism [43,44].

(4) Structural Robustness and Long-Term Stability Mechanisms

During 30 h stability testing at 240 °C, the catalyst maintains a methane conversion rate of 98.2 ± 0.5%, with post-cycling XRD showing negligible variation in (001) peak FWHM (0.35° ± 0.02°) and crystallite size (12.3 ± 0.5 nm). This stability originates from: (1) the rigid mesoporous m-Al_2_O_3_ framework suppressing Pt sintering; (2) topological confinement by layered MnO_2_ alleviating redox-induced lattice stress; and (3) strong Pt-Mn interactions (XPS binding energy shift ΔE = 0.8 eV) preventing active component leaching. Consistent O_2_-TPD and H_2_-TPR profiles (peak temperature drift < 5 °C) further validate the self-healing capability of the oxygen cycling network. The consistency between Raman-derived O_v_ regeneration rates and O_2_-TPD/activity data underscores the robustness of this methodology for probing dynamic oxygen cycling in confined catalysts.

A comprehensive comparison of the catalytic performance of our Pt–MnO_2_/Al_2_O_3_ catalysts with the representative literature systems is provided in Table 6.

## 4. Conclusions

This study establishes a multidimensional synergy in 0.5 wt% Pt-MnO_2_/Al_2_O_3_ catalysts for efficient low-temperature methane combustion, driven by the following:

(1) Mesoporous Al_2_O_3_ confinement enables sub-nanometer Pt dispersion (<2 nm) and enhances methane/oxygen adsorption. Coupled with reduced MnO_2_ crystallite size and lattice contraction (via Pt^2+^/Pt^4+^ doping), this architecture accelerates redox kinetics and lowers reduction activation energy.
(2) The PtO-MnPt_3_O_6_ heterointerface promotes a dual-functional mechanism: PtO activates gaseous O_2_ dissociation, while MnPt_3_O_6_ facilitates dynamic lattice oxygen migration. This synergy elevates oxygen vacancy regeneration rates by 2.3-fold higher, achieving a record-low T_90_ = 236 °C with 0.5 wt% Pt loading (Δ28 °C vs. undoped catalyst).
(3) Strong Pt-Mn bonding and mesoporous confinement suppress sintering and metal leaching, maintaining 98.2% methane conversion over 300 h at 240 °C. With 0.5 wt% Pt loading, this catalyst demonstrates scalable potential for industrial low-concentration methane elimination. The proposed “confinement–oxygen vacancy–interface” mechanism provides a universal framework for designing robust heterogeneous catalysts for hydrocarbon combustion.

## Figures and Tables

**Table 1:** Composition, nominal Pt loading, actual Pt loading (by ICP-OES), and sample codes of M_1_-MnO_2_/Al_2_O_3_ catalysts.

Sample	Mn/Citric Acid Molar Ratio	Nominal Metal Content (wt%)	Actual Metal Content by ICP-OES (wt%)
0 wt% Pt-MnO_2_/m-Al_2_O_3_	10:1	0	–
0.5 wt% Pt-MnO_2_/m-Al_2_O_3_	10:1	0.5	0.48 ± 0.02
1.0 wt% Pt-MnO_2_/m-Al_2_O_3_	10:1	1.0	0.96 ± 0.03
1.0 wt% Co-MnO_2_/m-Al_2_O_3_	10:1	1.0	0.94 ± 0.03 (Co)

**Table 2:** Crystallite size, specific surface area, pore volume, and yield of the synthesized catalysts.

Sample	M_1_O_x_-MnO_2_ Crystalline Size (nm)	SSA (m^2^ g^−1^)	Pore Volume (cm^3^ g^−1^)	Average Pore Diameter (nm)	Yield * (%)
0 wt% Pt-MnO_2_/m-Al_2_O_3_	15.9	20.3	0.425	19.7	51.2
0.5 wt% Pt-MnO_2_/m-Al_2_O_3_	15.5	27.6	0.334	20.7	47.2
1.0 wt% Pt-MnO_2_/m-Al_2_O_3_	12.7	28.1	0.387	27.1	48.1
1.0 wt% Co-MnO_2_/m-Al_2_O_3_	9.2	24.4	0.347	23.1	48.4

*SSA = Specific surface area determined by the BET method.* * Yield was calculated as the mass ratio of the active components (MnO_2_ and Pt/Co) in the final catalyst to the total mass of Mn and Pt/Co metals in the precursors (see Section 2.1). Crystallite sizes of M_1_O_x_–MnO_2_ phases were calculated through Williamson–Hall from the half width of the (001) diffraction peak (2θ ≈ 12.3°) in the XRD patterns. Pt crystallite sizes could not be estimated from XRD because no Pt diffraction peaks were observed, consistent with the sub-nanometer Pt dispersion confirmed by HAADF-STEM. Surface areas were calculated by the multi-BET method.

**Table 3:** Physicochemical properties of M_1_O_x_-MnO_2_ doped with different precious metals.

Sample	First Peak of TPR (°C)	O_latt_/at.%	Mn^3+^/at.%	H_2_ Consumption (mmol/g)	Catalytic Activity (°C)	
					T_50_	T_90_
0 wt% Pt-MnO_2_/m-Al_2_O_3_	303	58.78	75.22	8.09	241	264
0.5 wt% Pt-MnO_2_/m-Al_2_O_3_	287	71.01	76.63	8.53	224	236
1.0 wt% Pt-MnO_2_/m-Al_2_O_3_	273	74.99	79.87	8.67	222	229
1.0 wt% Co-MnO_2_/m-Al_2_O_3_	292	66.51	75.93	8.45	231	251

**Table 4:** Total amount of desorbed O_2_ and corresponding peak temperatures. The 0 wt% Pt sample is the bare MnO_2_/Al_2_O_3_ support (metal-free reference).

Catalyst	O_2_ Desorption (μmol/g)	Peak Temperature (°C)	O_2_ Release Increase (%)
0 wt% Pt-MnO_2_/m-Al_2_O_3_	120 ± 5	219	–
0.5 wt% Pt-MnO_2_/m-Al_2_O_3_	185 ± 6	213	+54
0.5 wt% Pt (spent, after 300 h)	176 ± 6	216	+47
1.0 wt% Pt-MnO_2_/m-Al_2_O_3_	210 ± 7	206	+75
1.0 wt% Co-MnO_2_/m-Al_2_O_3_	155 ± 5	216	+29

**Table 6:** Comparison of catalytic performance for methane oxidation over various Pt-based and related catalysts.

Catalyst	Pt Loading (wt%)	Performance Metric (Conversion)	T90 (°C)	Reaction Conditions (CH_4_ Conc., GHSV)	Active Site Morphology	Ref.
0.5 wt% Pt-MnO_2_/m-Al_2_O_3_	0.5	90%	236	1000 ppm, 30,000 mL·g^−1^·h^−1^	Sub-nm Pt clusters + Mn^3+^-O_v_	This work
1.0 wt% Pt-MnO_2_/m-Al_2_O_3_	1.0	90%	229	1000 ppm, 30,000 mL·g^−1^·h^−1^	Sub-nm Pt clusters + Mn^3+^-O_v_	This work
Pt/MnO_2_	1.0	90%	280	1000 ppm, 20,000 h^−1^	Atomically dispersed Pt	[8]
Pt/Al_2_O_3_	1.0	90%	~350	1000 ppm, 30,000 h^−1^	Pt nanoparticles (4–6 nm)	[4]
MnO_x_-Ni/MgAl_2_O_4_	0 (Ni: 10)	90%	~320	1% CH_4_, 42,000 h^−1^	Mn^4+^/Mn^3+^ redox pairs	[4]
Pt/CeO_2_	1.0	90%	310	0.5% CH_4_, 60,000 mL·g^−1^·h^−1^	Pt nanoparticles	[34]

## Data Availability

The original contributions presented in this study are included in the article. Further inquiries can be directed to the corresponding authors.

## References

[1] Akbari E., Alavi S.M., Rezaei M., Larimi A. (2021). Preparation and evaluation of A/BaO-MnO_X_ catalysts (A: Rh, Pt, Pd, Ru) in lean methane catalytic combustion at low temperature. Int. J. Energy Res..

[2] Cao Y., Su T., Ding Y., Song W., Yang L., Liu F., Zhai C. (2025). Enhanced combustion stability of low-concentration methane though a flame buffer zone in a variable pore-density porous media burner. Appl. Therm. Eng..

[3] Anić M., Radić N., Grbić B., Dondur V., Damjanović L., Stoychev D., Stefanov P. (2011). Catalytic activity of Pt catalysts promoted by MnO for n-hexane oxidation. Appl. Catal. B Environ..

[4] Varbar M., Alavi S.M., Rezaei M., Akbari E. (2022). Lean methane catalytic combustion over the mesoporous MnO_x_-Ni/MgAl_2_O_4_ catalysts: Effects of Mn loading. Int. J. Hydrogen Energy.

[5] Lee S., Seo J., Jung W. (2016). Sintering-resistant Pt@CeO_2_ nanoparticles for high-temperature oxidation catalysis. Nanoscale.

[6] Wang L., Hao L., Qi W., Huo X., Xue L., Liu Y., Zhang Q., Lin J. (2020). A sensitive Salmonella biosensor using platinum nanoparticle loaded manganese dioxide nanoflowers and thin-film pressure detector. Sens. Actuators B Chem..

[7] Xu Z., He X., Luo R., Zhang C., Zhang Z., Ren P., Zhang J., Chen X., Liu Y. (2025). PM@DCm nanohybrid-mediated pleiotropic antigen presentation for enhanced melanoma immunotherapy. Nano Today.

[8] Zhang H., Sui S., Zheng X., Cao R., Zhang P. (2019). One-pot synthesis of atomically dispersed Pt on MnO_2_ for efficient catalytic decomposition of toluene at low temperatures. Appl. Catal. B Environ..

[9] Qin Y., Wang H., Dong C., Qu Z. (2019). Evolution and enhancement of the oxygen cycle in the catalytic performance of total toluene oxidation over manganese-based catalysts. J. Catal..

[10] Wu Y., He C., Zhang W. (2022). “Capture-Backdonation-Recapture” Mechanism for Promoting N_2_ Reduction by Heteronuclear Metal-Free Double-Atom Catalysts. J. Am. Chem. Soc..

[11] Yu J.S., Park J.M., Kwon J.H., Park K.S., Choung J.W., Park M.-J., Bae J.W. (2023). Roles of Al_2_O_3_ coating layer on an ordered mesoporous Ni/m-Al_2_O_3_ for combined steam and CO_2_ reforming with CH_4_. Fuel.

[12] Jameei Moghaddam N., Akbari N., Nandy S., Chae K.H., Najafpour M.M. (2023). Effect of Different Metal Ions between Nanolayers of Manganese Oxide for Water Oxidation Reaction under Acidic Conditions. J. Phys. Chem. C.

[13] Mohanty A., Viet C.D., Roger A.-C., Adam A., Mertz D., Baaziz W., Janowska I. (2021). Structural impact of carbon nanofibers/few-layer-graphene substrate decorated with Ni for CO_2_ methanation via inductive heating. Appl. Catal. B Environ..

[14] Li Y., Chen T., Zhao S., Wu P., Chong Y., Li A., Zhao Y., Chen G., Jin X., Qiu Y. (2022). Engineering Cobalt Oxide with Coexisting Cobalt Defects and Oxygen Vacancies for Enhanced Catalytic Oxidation of Toluene. ACS Catal..

[15] Gu H., Lan J., Hu H., Jia F., Ai Z., Zhang L., Liu X. (2023). Surface oxygen vacancy-dependent molecular oxygen activation for propane combustion over α-MnO_2_. J. Hazard. Mater..

[16] Luo B., Zhang C., Ling X., Mukherjee S., Jia G., Xie J., Jia X., Liu L., Baulin E.F., Luo Y. (2023). Cryo-EM reveals dynamics of Tetrahymena group I intron self-splicing. Nat. Catal..

[17] Wu T., Rankin D.M., Golovko V.B. (2024). Electrochemical Oxidation of Low-Concentration Methane on Pt/Pt and Pt/CP under Ambient Conditions. ACS Omega.

[18] Meng F., Tang X., Kadja G.T., Yi H., Zhao S., Wu W., Zhang Y., Gao F., Yu Q. (2025). A systematic review with improving activity and stability in VOCs elimination by oxidation of noble metals: Starting from active sites. Sep. Purif. Technol..

[19] Sun Q., Zhang H., Fan Y., Bian L., Peng Q., Liu B. (2023). Regulating the electronic structure of Pd nanoparticles through metal alloy–support interactions for enhanced hydrogen generation. Renew. Energy.

[20] Wang R., Li J. (2009). OMS-2 Catalysts for Formaldehyde Oxidation: Effects of Ce and Pt on Structure and Performance of the Catalysts. Catal. Lett..

[21] Li L., Jing F., Yan J., Jing J., Chu W. (2017). Highly effective self-propagating synthesis of CeO_2_-doped MnO_2_ catalysts for toluene catalytic combustion. Catal. Today.

[22] Li D., Li W., Deng Y., Wu X., Han N., Chen Y. (2016). Effective Ti Doping of δ-MnO_2_ via Anion Route for Highly Active Catalytic Combustion of Benzene. J. Phys. Chem. C.

[23] Schulz H., Stark W.J., Maciejewski M., Pratsinis S.E., Baiker A. (2003). Flame-made nanocrystalline ceria/zirconia doped with alumina or silica: Structural properties and enhanced oxygen exchange capacity. J. Mater. Chem..

[24] Najafpour M.M., Isaloo M.A., Ghobadi M.Z., Amini E., Haghighi B. (2014). The effect of different metal ions between nanolayers of manganese oxide on water oxidation. J. Photochem. Photobiol. B Biol..

[25] Zhu W., Wu Z., Foo G.S., Gao X., Zhou M., Liu B., Veith G.M., Wu P., Browning K.L., Lee H.N. (2017). Taming interfacial electronic properties of platinum nanoparticles on vacancy-abundant boron nitride nanosheets for enhanced catalysis. Nat. Commun..

[26] Fan X., Xu P., Li Y.C., Zhou D., Sun Y., Nguyen M.A.T., Terrones M., Mallouk T.E. (2016). Controlled Exfoliation of MoS_2_ Crystals into Trilayer Nanosheets. J. Am. Chem. Soc..

[27] Chen S., Li L., Hu W., Huang X., Li Q., Xu Y., Zuo Y., Li G. (2015). Anchoring High-Concentration Oxygen Vacancies at Interfaces of CeO_2_–x/Cu toward Enhanced Activity for Preferential CO Oxidation. ACS Appl. Mater. Interfaces.

[28] Wu P., Wu Y., Chen L., He J., Hua M., Zhu F., Chu X., Xiong J., He M., Zhu W. (2020). Boosting aerobic oxidative desulfurization performance in fuel oil via strong metal-edge interactions between Pt and h-BN. Chem. Eng. J..

[29] Radic N., Grbic B., Terlecki B.A. (2004). Kinetics of deep oxidation of n-hexane and toluene over Pt/Al_2_O_3_ catalysts: Platinum crystallite size effect. Appl. Catal. B Environ..

[30] He H., Lin X., Li S., Wu Z., Gao J., Wu J., Wen W., Ye D., Fu M. (2018). The key surface species and oxygen vacancies in MnOx-CeO_2_ toward repeated soot oxidation. Appl. Catal. B Environ..

[31] Lu Y., Li C., Du X., Zhu Y., Zhang Y., Li S. (2020). Catalytic oxidation of toluene over MnO_2_ catalysts with different Mn (II) precursors and the study of reaction pathway. Fuel.

[32] Hou J., Li Y., Liu L., Ren L., Zhao X. (2013). Effect of giant oxygen vacancy defects on the catalytic oxidation of OMS-2 nanorods. J. Mater. Chem. A.

[33] Wang J., Li J., Jiang C., Zhou P., Zhang P., Yu J. (2017). The effect of manganese vacancy in birnessite-type MnO_2_ on room-temperature oxidation of formaldehyde in air. Appl. Catal. B Environ..

[34] Peng R., Sun X., Li S., Chen L., Fu M., Wu J., Ye D. (2016). Shape effect of Pt/CeO_2_ catalysts on the catalytic oxidation of toluene. Chem. Eng. J..

[35] Zhu L., Wang J., Rong S., Wang H., Zhang P. (2017). Cerium modified birnessite-type MnO_2_ for gaseous formaldehyde oxidation at low temperature. Appl. Catal. B Environ..

[36] Genuino H.C., Dharmarathna S., Njagi E.C., Mei M.C., Suib S.L. (2012). Gas-Phase Total Oxidation of Benzene, Toluene, Ethylbenzene, and Xylenes Using Shape-Selective Manganese Oxide and Copper Manganese Oxide Catalysts. J. Phys. Chem. C.

[37] Sun H., Liu Z., Chen S., Quan X. (2015). The role of lattice oxygen on the activity and selectivity of the OMS-2 catalyst for the total oxidation of toluene. Chem. Eng. J..

[38] Yang W., Su Z., Xu Z., Yang W., Peng Y., Li J. (2020). Comparative study of α-, β-, γ- and δ-MnO_2_ on toluene oxidation: Oxygen vacancies and reaction intermediates. Appl. Catal. B Environ..

[39] Wu Y., Feng R., Song C., Xing S., Gao Y., Ma Z. (2017). Effect of reducing agent on the structure and activity of manganese oxide octahedral molecular sieve (OMS-2) in catalytic combustion of o-xylene. Catal. Today.

[40] Li L., Luo J., Liu Y., Jing F., Su D., Chu W. (2017). Self-Propagated Flaming Synthesis of Highly Active Layered CuO-δ-MnO_2_ Hybrid Composites for Catalytic Total Oxidation of Toluene Pollutant. ACS Appl. Mater. Interfaces.

[41] Bendahou K., Cherif L., Siffert S., Tidahy H., Benaïssa H., Aboukaïs A. (2008). The effect of the use of lanthanum-doped mesoporous SBA-15 on the performance of Pt/SBA-15 and Pd/SBA-15 catalysts for total oxidation of toluene. Appl. Catal. A Gen..

[42] Liu S., Wu X., Liu W., Chen W., Ran R., Li M., Weng D. (2016). Soot oxidation over CeO_2_ and Ag/CeO_2_: Factors determining the catalyst activity and stability during reaction. J. Catal..

[43] Peng R., Li S., Sun X., Ren Q., Chen L., Fu M., Wu J., Ye D. (2018). Size effect of Pt nanoparticles on the catalytic oxidation of toluene over Pt/CeO_2_ catalysts. Appl. Catal. B Environ..

[44] Zhao S., Li K., Jiang S., Li J. (2016). Pd–Co based spinel oxides derived from pd nanoparticles immobilized on layered double hydroxides for toluene combustion. Appl. Catal. B Environ..

